# Mice lacking EFA6C/Psd2, a guanine nucleotide exchange factor for Arf6, exhibit lower Purkinje cell synaptic density but normal cerebellar motor functions

**DOI:** 10.1371/journal.pone.0216960

**Published:** 2019-05-16

**Authors:** Shintaro Saegusa, Masahiro Fukaya, Wataru Kakegawa, Manabu Tanaka, Osamu Katsumata, Takeyuki Sugawara, Yoshinobu Hara, Makoto Itakura, Tadashi Okubo, Toshiya Sato, Michisuke Yuzaki, Hiroyuki Sakagami

**Affiliations:** 1 Department of Anatomy, Kitasato University School of Medicine, Sagamihara, Kanagawa, Japan; 2 Department of Physiology, Keio University School of Medicine, Tokyo, Japan; 3 Bio-imaging Center, Kitasato University School of Medicine, Sagamihara, Kanagawa, Japan; 4 Department of Biochemistry, Kitasato University School of Medicine, Sagamihara, Kanagawa, Japan; 5 Department of Laboratory Animal Science, Kitasato University School of Medicine, Sagamihara, Kanagawa, Japan; University of South Alabama, UNITED STATES

## Abstract

ADP ribosylation factor 6 (Arf6) is a small GTPase that regulates various neuronal events including formation of the axon, dendrites and dendritic spines, and synaptic plasticity through actin cytoskeleton remodeling and endosomal trafficking. EFA6C, also known as Psd2, is a guanine nucleotide exchange factor for Arf6 that is preferentially expressed in the cerebellar cortex of adult mice, particularly in Purkinje cells. However, the roles of EFA6C in cerebellar development and functions remain unknown. In this study, we generated global EFA6C knockout (KO) mice using the CRISPR/Cas9 system and investigated their cerebellar phenotypes by histological and behavioral analyses. Histological analyses revealed that EFA6C KO mice exhibited normal gross anatomy of the cerebellar cortex, in terms of the thickness and cellularity of each layer, morphology of Purkinje cells, and distribution patterns of parallel fibers, climbing fibers, and inhibitory synapses. Electron microscopic observation of the cerebellar molecular layer revealed that the density of asymmetric synapses of Purkinje cells was significantly lower in EFA6C KO mice compared with wild-type control mice. However, behavioral analyses using accelerating rotarod and horizontal optokinetic response tests failed to detect any differences in motor coordination, learning or adaptation between the control and EFA6C KO mice. These results suggest that EFA6C plays ancillary roles in cerebellar development and motor functions.

## Introduction

ADP ribosylation factor 6 (Arf6) is a small GTPase that regulates actin cytoskeleton remodeling and vesicular transport between the plasma membrane and endosomes [1–3]. In the mammalian brain, Arf6 mediates a variety of neuronal processes related to cell shape, motility and responsiveness, including the maintenance of structural integrity of the neuroepithelium [4], neuronal migration [5, 6], formation of the axon [7] and dendrites [8], maturation and maintenance of dendritic spines [9–11], trans-regulation of oligodendrocyte differentiation [12], axonal transport [13], recycling of synaptic vesicles [14] and hippocampal long-term depression [15]. The diverse neuronal functions of Arf6 are facilitated by strict spatiotemporal regulation of the GDP/GTP cycle of Arf6 in neurons by two types of regulatory proteins: guanine nucleotide exchange factors (GEFs) that facilitate the exchange of GDP for GTP, and GTPase activating proteins (GAPs) that enhance GTP hydrolysis. Several GEFs were previously identified to activate Arf6, including the EFA6/PSD (exchange factor for Arf6/pleckstrin and Sec7 domain-containing protein), BRAG/IQSEC (brefeldin A-resistant Arf-GEF/IQ motif and Sec7 domain-containing protein) and cytohesin families [1, 2]

The EFA6/PSD family comprises EFA6A/PSD1 [16, 17], EFA6B/PSD4 [18], EFA6C/PSD2 [19] and EFA6D/PSD3 [20], which are generated from distinct genes and function primarily as an Arf6-specific GEF [16, 21, 22]. They are structurally characterized by a conserved domain organization consisting of a central catalytic Sec7 domain, an adjacent pleckstrin homology (PH) domain responsible for interaction with the plasma membrane and F-actin [18, 23], and a C-terminal region containing a coiled coil motif that mediates protein-protein interaction and GEF-independent actin cytoskeleton remodeling [16, 17, 24, 25]. In the adult mouse brain, EFA6A, EFA6C and EFA6D are abundantly expressed with distinct expression patterns. EFA6A is expressed predominantly in the forebrain and localized to the plasma membrane, postsynaptic density and endosomes in the dendritic shaft and spines in hippocampal neurons [17, 25, 26]. The EFA6A-Arf6 pathway was shown to regulate dendritic formation [26, 27], maturation and maintenance of dendritic spines [9, 11], and directionality of axonal transport [28] in primary cultured neurons. EFA6D is widely expressed throughout the brain and is localized to various subcellular compartments in hippocampal neurons, including cell bodies, dendritic shafts and spines, axons and presynaptic terminals [20, 29]. A single nucleotide polymorphism in the human EFA6D gene was reportedly associated with alcohol drinking behaviors and neuronal activity in the prefrontal cortex during the go/no-go executive control task, suggesting modulatory roles of EFA6D in addictive and cognitive behaviors [30]. On the other hand, EFA6C is unique in that it is expressed predominantly in the cerebellar cortex, particularly in Purkinje cells [19]. However, the physiological significance of individual EFA6 members in the brain is still unknown at the whole animal level.

The cerebellum is a brain region that is engaged in motor coordination and learning with a uniform three-layered cortical structure consisting of the molecular layer, Purkinje cell layer and granular layer [31, 32]. Purkinje cells are the sole output neurons of the cerebellar cortex, with an extremely elaborate dendritic tree studded with numerous dendritic spines, where asymmetric excitatory synapses are formed with parallel fibers from granule cells and climbing fibers from the inferior olive at the distal and proximal dendritic segments, respectively. Owing to their unique morphology and relatively simple synaptic connections [31, 32], Purkinje cells have been extensively studied as a model for neuronal morphogenesis, synaptogenesis and synaptic plasticity.

To examine the physiological role of EFA6C in cerebellar development and functions, we generated mice lacking EFA6C using the CRISPR/Cas9 system and examined their cerebellar phenotypes by histological and behavioral analyses.

## Materials and methods

### Ethnics statement

All animal experiments were carried out in accordance with the guideline of the National Institutes of Health, and the Ministry of Education, Culture, Sports, Science and Technology (MEXT) of Japan and approved by the Animal Experimentation and Ethics Committee of the Kitasato University School of Medicine (Permission number: 2017–146). All efforts were made to minimize animal distress and to reduce the number of animals used.

### Generation of global EFA6C knockout (KO) mice

The guide RNA (gRNA) consisting of a T7 promoter sequence (5’-TAATACGACTCACTATAGG-3’) followed by a 23-nucleotide target sequence for exon 1 of the EFA6C gene including the protospacer adjacent motif (PAM, underlined) (5’-GAAGACCTTAGAGGGTACCATGG-3’) and 80-nucleotide constant region of CRISPR RNA (crRNA) and trans-activating crRNA (tracrRNA) was synthesized commercially (Thermo Fisher Scientific, Carlsbad, CA). The fertilized zygotes were generated by *in vitro* fertilization using C57BL/6J female and male mice, and electroporated with the gRNA and Cas9 protein (Integrated DNA Technologies, Coralville, IA) using platinum plate electrodes on tempered glass (LF501PT-1, BEX, Tokyo, Japan) connected to CUY21EDIT II (BEX) under the pulse conditions (30V, 3 ms pulse duration, 97 ms interval, 7 repeats) described previously [33]. After incubation of the zygotes in Whitten’s medium for 24 h at 37°C and 5% CO_2_, the surviving two-cell-stage embryos were transferred into the oviducts of pseudo-pregnant female mice.

To check CRISPR/Cas9-mediated mutation of the EFA6C gene, genomic DNA was extracted from tail or ear tissues and subjected to PCR using primers to amplify the exon 1 region including the gRNA target sequence: forward (5’- ACCGACATGGATGAAGAGAAGCTC-3’) and reverse (5’-TCCTCCTCTGCTGGTCCGCTTCTC-3’). The PCR amplicons were visualized by agarose gel electrophoresis, subcloned into pGEM-T Easy vectors (Promega, Madison, WI) and sequenced using the BigDye terminator Cycle Sequencing Kit v3.1 and ABI 3100 Genetic Analyzer (Applied Biosystems, Foster City, CA). The male founder mice were crossed to wild-type C57BL/6J female mice to generate heterozygous offspring.

Adult mice (less than 5 per cage) and pregnant mice (1 per cage) were housed and maintained under barrier conditions in an air-conditioned room (about 20–24°C) under 12-h light/dark cycle with free access to food and water. In all experiments, wild-type mice generated by intercrossing heterozygous male and female mice were used as control animals.

### Immunoblotting

Cerebella were freshly taken from three male 10–12-week-old mice of each genotype that had been sacrificed by cervical dislocation under the inhalation anesthesia with 4–5% isoflurane, and homogenized by sonication in a solution consisting of 125 mM TrisHCl (pH 6.8), 4% sodium dodecyl sulfate (SDS), 20% glycerol, and 100 mM dithiothreitol (DTT), and incubated for 10 min at 50°C. The protein concentration of each lysate was determined using the bicinchoninic acid assay (Pierce BCA protein assay kit, Cat. No. 23225, Thermo Fisher Scientific). HeLa cells were transfected with a mammalian expression vector carrying N-terminally FLAG epitope-tagged mouse EFA6C (pCAGGS-FLAG-mEFA6C) [19] using Lipofectamine 2000 (Thermo Fisher Scientific). One day after transfection, cells were harvested with a solution consisting of 125 mM TrisHCl (pH 6.8), 4% SDS, 20% glycerol and 10% 2-mercaptoethanol, and boiled for 5 min. Total lysates of cerebellar samples (10 μg) and HeLa cells over-expressing FLAG-mEFA6C were subjected to immunoblotting with antibodies against EFA6A [17], EFA6C [19] (Cat. No. 17404-1-AP, ProteinTech, Rosemont, IL), EFA6D [29], BRAG1 [34], BRAG2 [35], BRAG3 [36], cytohesin-2 [37], Arf6 [38], calbindin (Cat. No. Calbindin-Rb-Se-1, RRID: AB_2571568, Frontier Institute, Ishikari, Japan) and mGluR1a (Cat. No. mGluR1a-Rb-Af811, PRID:AB_2571799, Frontier Institute) as listed in Table 1. After incubation with peroxidase-conjugated species-specific secondary antibodies (1:5000, GE Healthcare, Tokyo, Japan) for 1 h, the blots were treated with a chemiluminescent detection reagent (Western Blotting Substrate Plus, Pierce, Rockford, IL) and the chemiluminescence was detected using an image analyzer (Amersham Imager 680, GE Healthcare). The optical density of each immunoreactive band was measured using the ImageQuant TL software (GE Healthcare). After the background density, which was taken from the area adjacent to the measured immunoreactive band, was subtracted, the value of each immunoreactive band was normalized to that of α-tubulin. Data were shown as means ± SEM of relative ratio to the wild-type control group set as 1.

**Table 1:** Primary antibodies used in this study.

Antibody	Immunogen	Host Species	Dilution	Source
EFA6A	Recombinant protein corresponding to amino acids 376–1024 of mouse EFA6A	rabbit polyclonal	0.25 μg/ml (IB)	Sakagami et al., 2007 [17]
EFA6C (N)	Recombinant protein corresponding to amino acids 1–136 at the N-terminus of mouse EFA6C	rabbit polyclonal	0.1 μg/ml (IB) 1 μg/ml (IHC)	Matsuya et al., 2005 [19]
EFA6C (C)	Recombinant protein corresponding to amino acids 418–771 at the C-terminus of human EFA6C	rabbit polyclonal	0.25 μg/ml (IB)	Proteintech, #17404-1-AP
EFA6D	Recombinant protein corresponding to amino acids 1–680 amino acids at the N-terminus of mouse EFA6D1/2b	guinea pig polyclonal	0.25 μg/ml (IB)	Fukaya et al., 2016 [29]
Cytohesin-2	Recombinant protein corresponding to amino acids 387–400 at the C-terminus of mouse cytohesin-2	guinea pig polyclonal	0.25 μg/ml (IB)	Ito et al., 2018 [37]
BRAG1	Recombinant protein corresponding to amino acids 1364–1479 at the C-terminus of mouse BRAG1/Iqsec2	rabbit polyclonal	0.25 μg/ml (IB)	Sakagami et al., 2008 [34]
BRAG2	Recombinant protein corresponding to amino acids 259–366 of mouse BRAG2/GEP100/Iqsec1 (NM_001134383)	guinea pig polyclonal	0.25 μg/ml (IB)	Sakagami et al., 2013 [35]
BRAG3	Recombinant protein corresponding to amino acids 1–293 at the N-terminus of rat synArfGEF/BRAG3/Iqsec3	rabbit polyclonal	0.25 μg/ml (IB)	Fukaya et al., 2011 [36]
Arf6	Synthetic peptide corresponding to 166–175 at the C-terminus of mouse Arf6	guinea pig polyclonal	0.25 μg/ml (IB)	Katsumata et al., 2017 [38]
Calbindin	Recombinant protein of full-length mouse calbindin	rabbit polyclonal	0.25 μg/ml (IB) 1 μg/ml (IF)	Frontier Institute, #Calbindin-Go-Af1040, PRID:AB_2532104
mGluR1a	Recombinant protein corresponding to amino acids 945–1127 of mouse GluR1a	rabbit polyclonal	0.25 μg/ml (IB)	Frontier Institute, #mGluR1a-Rb-Af811, PRID:AB_2571799
α-tubulin	Full length chicken α-tubulin purified from brain extracts	mouse monoclonal IgG1	0.058 μg/ml (IB)	Sigma-Aldrich, #T9026, clone: DM1A, PRID:AB_477593
VGluT1	Recombinant protein corresponding to amino acids 531–560 at the C-terminus of mouse VGulT1	rabbit polyclonal	1 μg/ml (IF)	Frontier Institute, #VGluT1-Rb-Af500, PRID:AB_2315558
VGluT2	Recombinant protein corresponding to amino acids 559–582 at the C-terminus of mouse VGulT2	guinea pig polyclonal	1 μg/ml (IF)	Frontier Institute, #VGluT2-GP-Af810, PRID:AB_2571621

Abbreviations: IB, immunoblotting; IF, immunofluorescence; IHC, immunohistochemistry.

### Histological analyses

Three male 10–12-week-old mice of each genotype were perfused through the vasculature via the left cardiac ventricle with 4% paraformaldehyde and 2% paraformaldehyde/2.5% glutaraldehyde in 0.1 M phosphate buffer (pH 7.2) for light and electron microscopic analyses, respectively.

Immunoperoxidase and immunofluorescence staining were performed as previously described [31]. For immunoperoxidase staining, 4–6 paraffin sections (5 μm thick) of brains from three male mice of each genotype were treated with 0.3% Triton X-100 in phosphate-buffered saline (PBS) for 15 min and 5% goat serum in PBS for 30 min, followed by the incubation overnight with rabbit anti-EFA6C IgG [19]. They were subsequently incubated with peroxidase-conjugated anti-rabbit IgG (Histofine Simple Stain MAX PO (R) kit, Nichirei, Tokyo, Japan) as a secondary antibody for 2 h. The immunoreaction was visualized in substrate solution containing 3,3’-diaminobenzidine and hydrogen peroxide (Liquid DAB+ Substrate Chromogen System, Cat. No. K3468, DAKO, Carpinteria, CA). For immunofluorescence, 4–6 parasagittal microslicer sections (50 μm thick) of cerebella from three male mice of each genotype were pre-treated with 0.3% Triton X-100 and 5% donkey serum, and incubated overnight with rabbit anti-calbindin (Frontier Institute) or rabbit anti-VGluT1 (Cat. No. VGluT1-Rb-Af500, RRID: AB_2571616, Frontier Institute) and guinea pig anti-VGluT2 (Cat. No. VGluT2-GP-Af810, RRID: AB_2571621, Frontier Institute) antibodies (Table 1). Immunoreactions were visualized with species-specific secondary antibodies conjugated with Alexa Fluor 488, 594 or 645 (Jackson ImmunoResearch Inc., West Grove, PA). Immunofluorescent images were taken by a confocal laser-scanning microscope (LSM 710; Carl Zeiss, Oberkochen, Germany) using a Plan-Apochromat 20x/0.8 objective lens (Carl Zeiss) with 1.5–2.0-fold digital zoom. The brightness and contrast of fluorescent images were subjected to minimal modification using the ZEN imaging software (Carl Zeiss) and Photoshop CS4 software (Adobe Systems, San Jose, CA). For morphological analysis, 3–5 parasagittal paraffin sections (5 μm thick) of brains from three male mice of each genotype were also stained with 0.1% cresyl violet solution.

Electron microscopic analyses were performed as previously described [39] with some modifications. Parasagittal sections (200 μm thick) of cerebella prepared with a microslicer (VT1000S; Leica, Nussloch, Germany) were post-fixed with 2% osmium tetroxide, dehydrated, and embedded in epoxy resin. Ultrathin sections of the cerebral cortex of lobules 4/5 were made at a thickness of 70 nm in the parasagittal plane on an ultra-microtome (Ultracut; Leica), and stained with 2% uranyl acetate and mixed lead solution. Thirty electron microscopic images of the middle one-third of the molecular layer with an area of 300 μm^2^ were randomly taken from three mice of each genotype using an electron microscope (H-7650; Hitachi, Tokyo, Japan). Asymmetric synapses were identified as synaptic contacts between parallel fiber terminals, which contain synaptic vesicles at a lower packaging density than climbing fiber terminals, and the Purkinje cell spine heads, which are observed as round or oval profiles containing thick electron-dense postsynaptic specialization and smooth endoplasmic reticulum without mitochondria or microtubules [40]. The largest diameter of the spine head was measured as the spine head width. The length of thick electron-dense postsynaptic specialization in the Purkinje cell spine was measured as the postsynaptic density length. The measurement was done in a blinded manner using the Image J software (NIH). To determine the percentage of free spines of Purkinje cells, approximately 100 spines from each mouse (WT, 330 spines; KO, 337 spines) were examined with several sets of serial electron microscopic images from three wild-type and EFA6C KO mice as described previously [39].

### Behavioral analyses

The rotarod test was performed as previously described with modification [41]. Briefly, the rotarod apparatus was programmed to accelerate from 4 rpm to 40 rpm over 4 min and then remain constant speed at 40 rpm for 1 min. Ten male mice aged 10–12 weeks from each genotype were placed on the rod rotating at 4 rpm and underwent three trials per day with a 30-min inter-trial interval for 5 consecutive days. The latency until mice fell off the rod or rotated all the way around the rod successively twice while clinging to the rod was recorded, and data from the three trials were averaged to give the daily score. One day before beginning the behavioral tests, mice were placed on the rod rotating at 4 rpm for 1 min to acclimate to walking on the rod.

Five male mice aged at 10–12 weeks from each genotype were subjected to a horizontal optokinetic response test as described previously [42, 43]. Briefly, mice were placed on a table with their heads fixed by a screw towards a checked-pattern screen, which was sinusoidally oscillated around the table by 15 degrees (peak-to-peak) at 0.33 Hz in light. The evoked eye movements were recorded by an infrared television camera system (Flea3 0.3 MP Mono; FLIR system, OR), and several cycles of the evoked eye movements without blinks and saccades were averaged every 10 min for 1 h, and the mean amplitudes were calculated by a modified Fourier analysis. The gain of the eye movement was calculated by dividing the peak-to-peak amplitude of eye movement by that of the screen oscillation at each time point.

### Statistical analyses

Data from histological and behavioral analyses were statistically analyzed using Student’s t-test and two-way repeated measures ANOVA, respectively. Significance was indicated by a p-value less than 0.05.

## Results

### Generation and characterization of EFA6C KO mice

**Fig 1:**
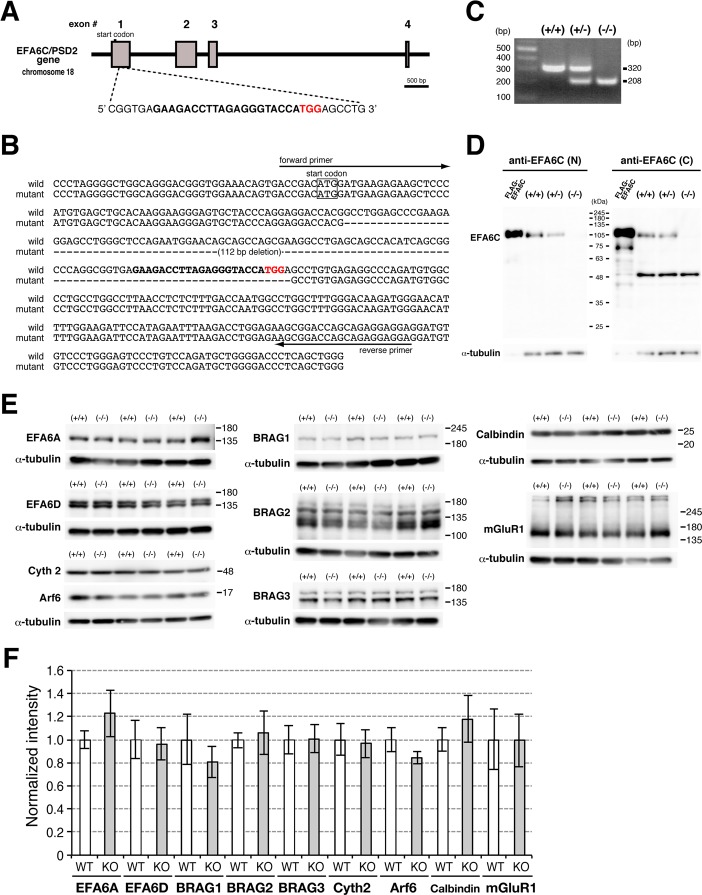
Generation and characterization of EFA6C KO mice. (A) Schematic illustration of the strategy to generate EFA6C KO mice. The gRNA sequence targeting exon 1 of the mouse EFA6C gene and the PAM sequence are shown in bold and red, respectively. (B) Sequence alignment of exon 1 in wild-type and mutant alleles. Note the deletion of 112 nucleotides in the mutant allele. The sequences of the target and PAM are shown in bold and red, respectively. (C) PCR genotyping using primers indicated in Fig 1B. The expected sizes of wild-type and mutant amplicons were 320 and 208 bp, respectively. The positions and sizes (bp) of DNA markers are indicated on the left. (D, E) Immunoblot analyses. The lysates of cerebella from three male mice of each genotype and HeLa cells exogenously expressing FLAG-EFA6C were subjected to immunoblotting with antibodies against EFA6C N-terminal region (EFA6C[N]), EFA6C C-terminal region (EFA6C[C]), EFA6A, EFA6D, BRAG1–3, cytohesin-2 (Cyth2), Arf6, calbindin and mGluR1. The same blots were reprobed with anti-α-tubulin IgG. Note that immunoreactive bands for EFA6C were completely absent in the cerebellar lysates of homozygous mice, and there were no apparent differences in the protein expression of Arf6 GEFs, Arf6, calbindin and mGluR1 in the cerebellar lysates between the two genotypes. The positions and sizes (kDa) of molecular weight markers are indicated on the right. (F) Quantification of the protein expression of Arf6 GEFs, Arf6, calbindin and mGluR1. Relative optical densities for immunoreactive bands normalized to those for α-tubulin are shown as mean ± SEM of relative ratio to the wild-type control group set as 1.

To investigate the physiological role of EFA6C *in vivo*, we generated EFA6C KO mice using the CRISPR/Cas9 system. The gRNA containing 20 nucleotides followed by a 3-nucleotides PAM sequence targeting exon 1 of the EFA6C gene (Fig 1A) was electroporated into fertilized zygotes together with the Cas9 protein. Among the ten founder mice carrying indels in the EFA6C gene, PCR and sequencing analyses revealed that six founder mice (5 males and 1 females) carried bi-allelic mutations around the target sequence, two mice (2 females) carried a mono-allelic mutation and the remaining two male mice were mosaic carrying more than two mutant alleles (S1 Fig). By crossing the male founder mice to wild-type C57BL/6J female mice, heterozygous offspring were generated, and a mouse line derived from the founder mice #10 (S1 Fig), in which 112 nucleotides had been deleted in the exon 1, was chosen for further investigation (Fig 1B and 1C). Homozygous mice grew normally without any abnormal neurological signs such as ataxic gait.

To assess the protein expression of EFA6C, cerebellar lysates from each genotype were immunoblotted with anti-EFA6C antibodies raised against the N-terminal 136 amino acids of mouse EFA6C (anti-EFA6C[N]) [19] or C-terminal 354 amino acids of human EFA6C (anti-EFA6C[C]). Both anti-EFA6C antibodies detected an immunoreactive band around 110 kDa, which was the same electrophoretic mobility as FLAG-EFA6C, in the cerebellar lysates of wild-type and heterozygous mice, with the expression level correlated with the allele number (EFA6C (anti-EFA6C[N])/α-tubulin: WT, 1.0 ± 0.05; Hetero (+/-), 0.62 ± 0.04; KO, 0.02 ± 0.004; EFA6C (anti-EFA6C[C]) /α-tubulin: WT, 1.0 ± 0.26; Hetero (+/-), 0.49 ± 0.02; KO, 0.03 ± 0.01, n = 3 of each genotype), whereas the band was undetectable in the those of EFA6C KO mice (Fig 1D). In addition, the anti-EFA6C(C) antibody detected a band around 50 kDa without differences in the immunoreactive intensity among the three genotypes (Fig 1D, WT, 1.0 ± 0.42; Hetero (+/-), 0.76 ± 0.10; KO, 0.74 ± 0.01, n = 3 of each genotype). No other bands corresponding to truncated EFA6C products were detectable in the cerebellar lysates of homozygous or heterozygous mice. Next, to examine the compensatory changes in the Arf6 pathway, the cerebellar lysates were immunoblotted with antibodies directed against Arf6 and other Arf6 GEFs such as EFA6A [16], EFA6D [20], BRAG1–3 [34, 36, 44] and cytohesin-2 [45]. There were no significant differences in the protein expression of Arf6 and other Arf6 GEFs in the cerebellar lysates between wild-type and EFA6C KO mice (Fig 1E and 1F). Further immunoblotting of cerebellar lysates with antibodies against calbindin and mGluR1a, which are cytoplasmic and postsynaptic proteins enriched in Purkinje cells [46, 47], respectively, failed to detect any quantitative differences in their protein expression levels between the two genotypes (Fig 1E and 1F).

**Fig 2:**
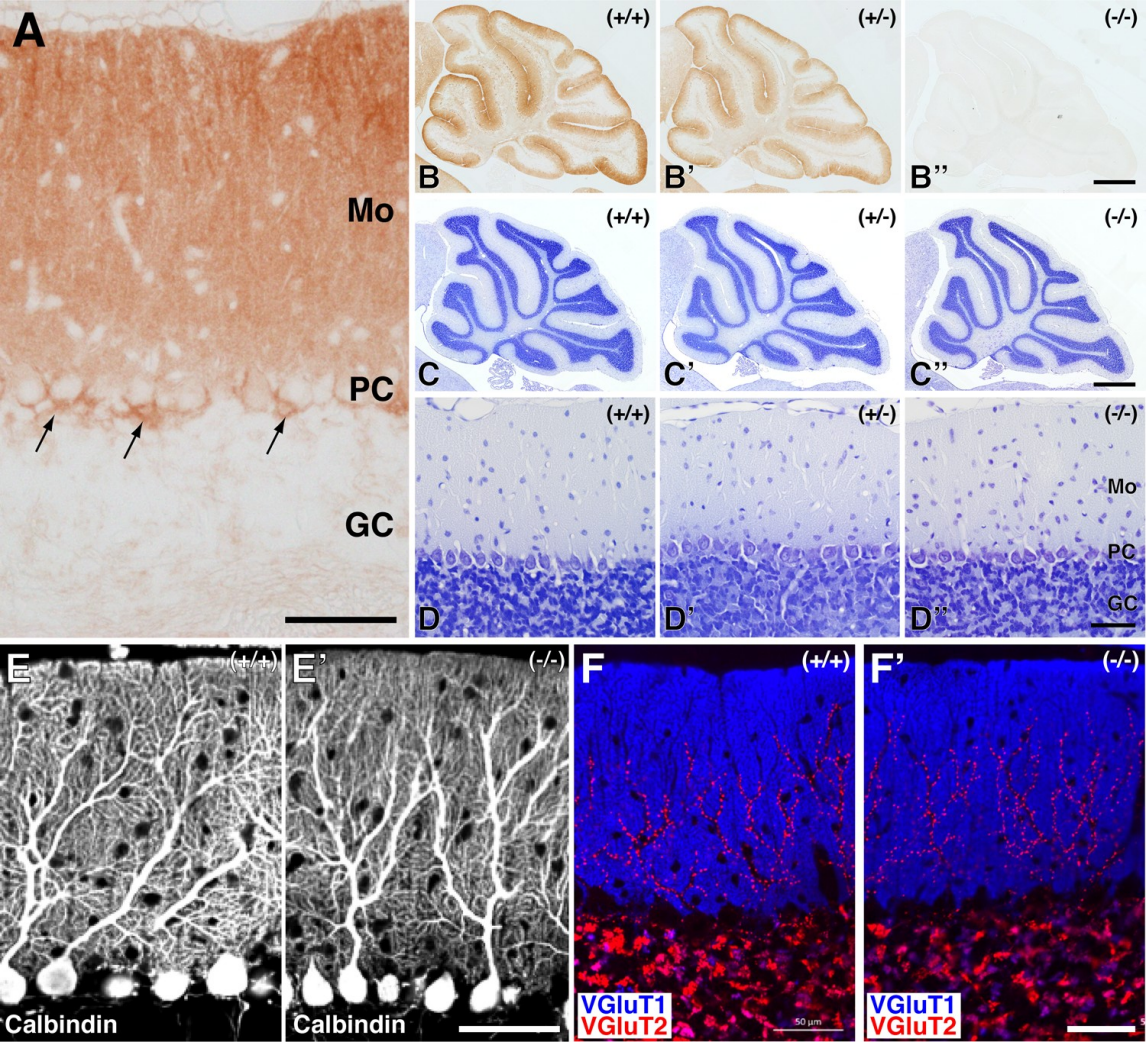
Histological analyses of the cerebellar cortex of wild-type and EFA6C KO mice. (A) Immunohistochemical localization of EFA6C in the wild-type mouse cerebellar cortex. Note the intense immunolabeling for EFA6C in the Purkinje cell bodies, molecular layer (Mo) and cerebellar pinceau (arrows). GC, the granular layer. (B–B”) Immunoperoxidase staining of sagittal sections of cerebella of 10–12-week-old wild-type (+/+) (B), heterozygous (+/-) (B’) and homozygous (-/-) (B”) mice with the anti-EFA6C(N) antibody. (C–D) Nissl staining of sagittal sections of cerebella from 10–12-week-old wild-type (C, D), heterozygous (C’, D’) and homozygous (C”, D”) mice. Note the lack of significant differences in the gross cerebellar structure among the three genotypes. (E, F) Immunofluorescence. Sagittal sections of cerebella from wild-type (WT, E, F) and EFA6C KO (E’, F’) mice aged at 10–12 weeks were subjected to immunostaining with antibodies against calbindin (E, E’), or VGluT1 (blue) and VGluT2 (red) (F, F’). Note the lack of apparent differences in the dendritic arborization of Purkinje cells (E, E’) and the immunoreactive pattern of VGluT1 and VGluT2 in the molecular layer (F, F’) between the two genotypes. PC, Purkinje cell layer. Scale bars, 50 μm in A, D”, E’ and F’; 500 μm in B” and C”.

Immunohistochemical analyses with the anti-EFA6C(N) antibody demonstrated that intense immunolabeling for EFA6C was detected in cell bodies and dendrites of Purkinje cells and the pinceau of basket cells in the wild-type cerebellar cortex (Fig 2A and 2B). In addition, faint but discrete immunolabeling was detected in the cerebellar glomeruli in the granular layer. In contrast, no immunolabeling was detectable in the cerebellar cortex of homozygous mice (Fig 2B”), further confirming the absence of EFA6C at the protein level in the cerebellum of EFA6C KO mice.

### Histological assessment

Nissl staining of parasagittal cerebellar sections failed to detect differences in the gross anatomy of the cerebellar cortex between wild-type, heterozygous and homozygous mice in terms of the formation of folia, the three-layered structural organization of the cerebellar cortex, and the thickness and cellularity of each layer (Fig 2C–2C” and 2D–2D”).

Immunofluorescence with an antibody against calbindin, a reliable marker to visualize the overall morphology of Purkinje cells [48, 49], revealed that the Purkinje cells of both wild-type and homozygous mice developed extensive dendritic arbors that were indistinguishable (Fig 2E and 2E’). Double immunofluorescence with antibodies against VGluT1 and VGluT2, which label axon terminals of parallel fibers from cerebellar granule cells and climbing fibers from inferior olivary neurons, respectively, revealed that there were no apparent qualitative differences between the two genotypes in the labeling pattern or intensity for VGluT1 and VGluT2 in the molecular layer (Fig 2F and 2F’), suggesting the normal development of the two major afferent fibers onto Purkinje cells. Further double immunofluorescence with antibodies against VGAT and PV, which label GABAergic axon terminals, and Purkinje cells and interneurons such as basket cells and stellate cells, respectively, did not detect any qualitative differences in the immunoreactive cell number or distribution pattern between the two genotypes (data not shown).

**Fig 3:**
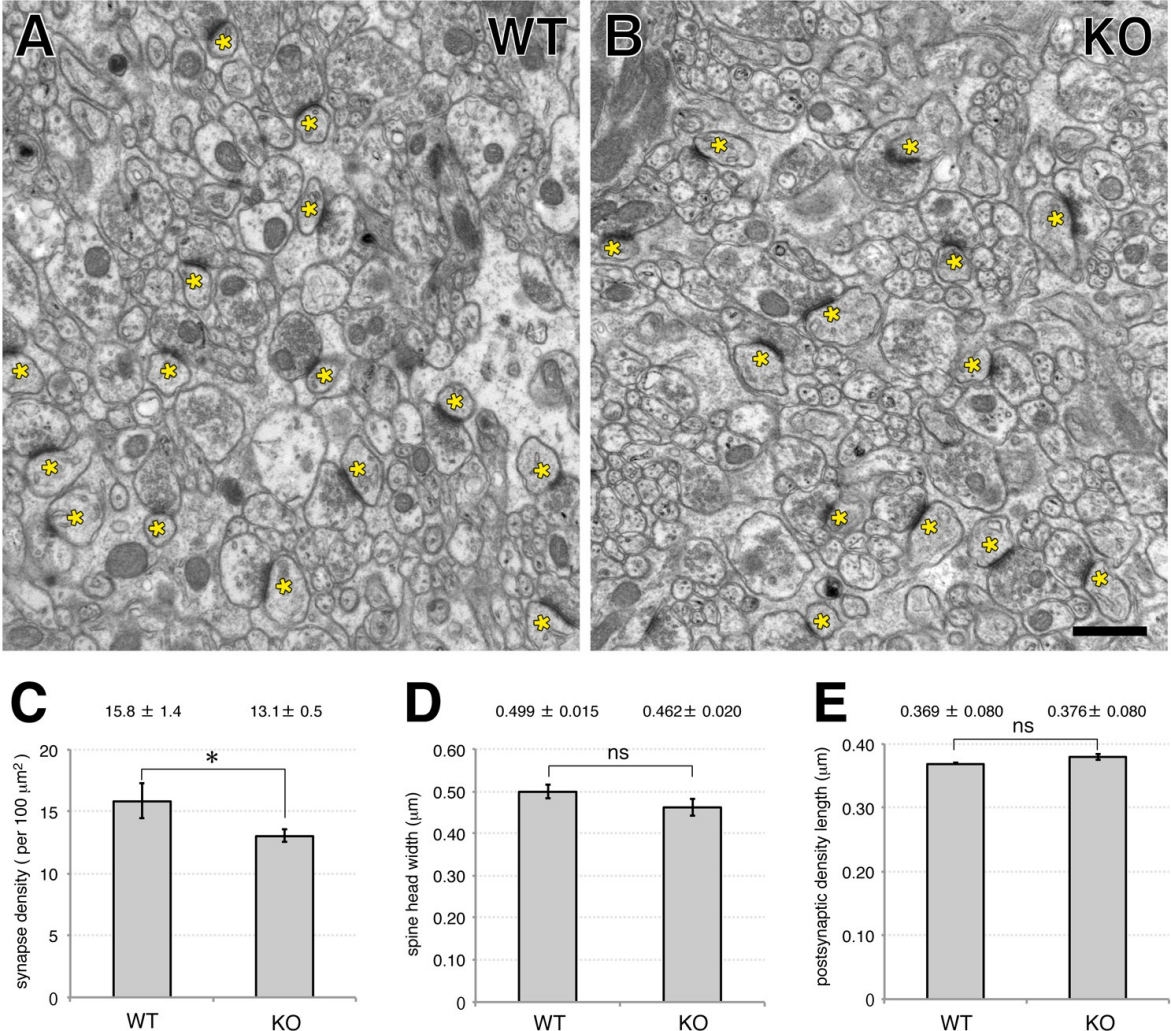
Ultrastructural analysis of the cerebellar molecular layer of wild-type and EFA6C KO mice. (A, B) Representative electron micrographs of the cerebellar molecular layer of 10–12-week-old wild-type (WT) (A) and EFA6C KO (B) mice. Asterisks indicate asymmetric synapses on Purkinje cell spines. (C–E) Quantification of the density of asymmetric synapses (C), spine head width (D) and length of postsynaptic density (E). Thirty images of the middle one-third of the molecular layer with an area of 300 μm^2^ were taken randomly from three male mice of each genotype, and examined in a blinded manner. Note the significantly lower density of asymmetric synapses in EFA6C KO mice compared with that in wild-type mice. Data are shown as mean ± SEM from three mice of each genotype. * p < 0.05; ns, not significant; Student’s t-test. Scale bar, 1 μm.

**Fig 4:**
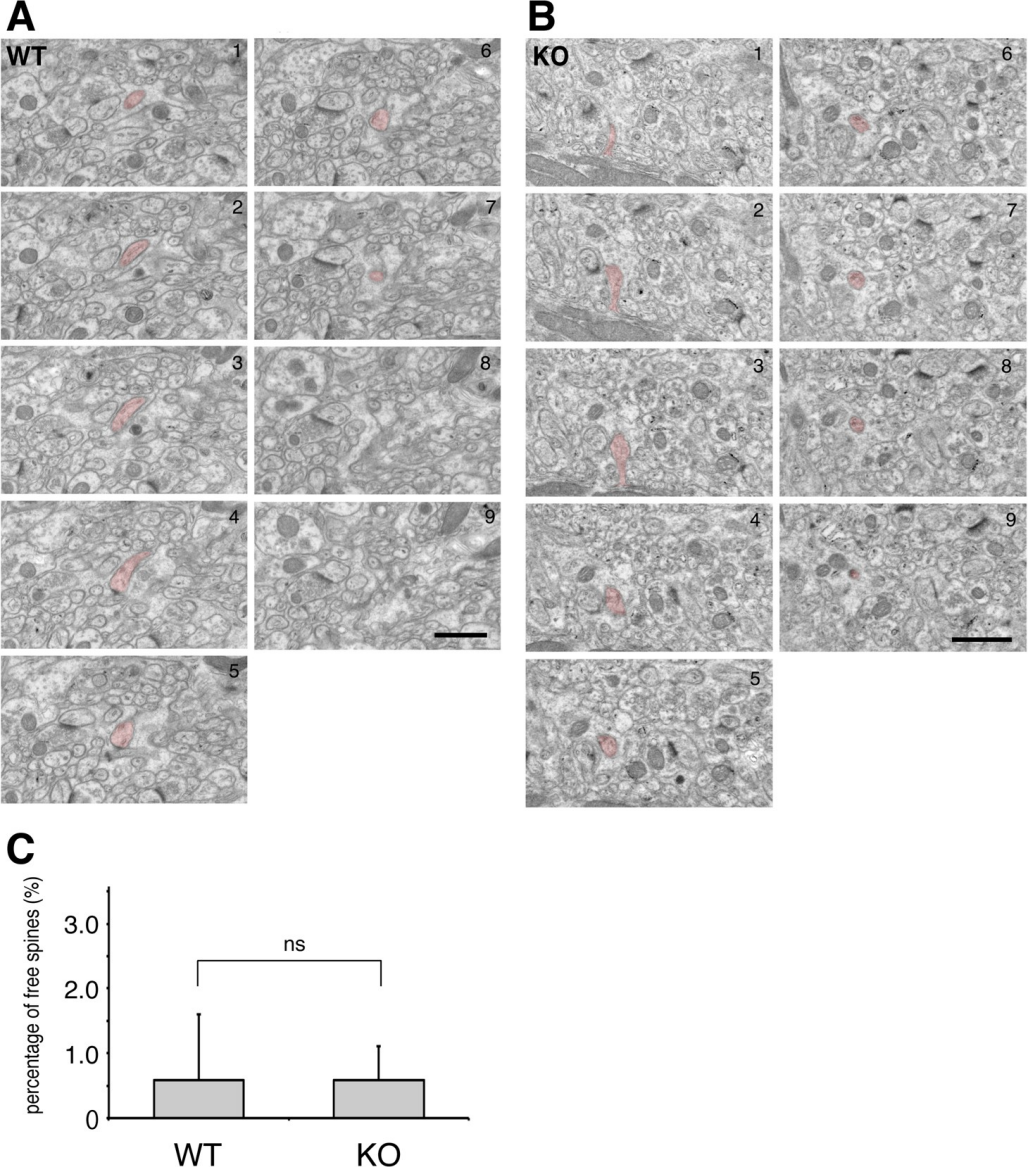
Serial electron micrographs of the molecular layer of wild-type and EFA6C KO mice. Representative examples of a free spine observed in the molecular layer of wild-type (WT, A) and EFA6C KO (B) mice are shown in red. (C) Quantification of the percentage of free dendritic spines showing the lack of significant differences in the incidence of free spines between the two genotypes. Data are shown as mean ± SEM from three mice of each genotype (WT, 330 spines; KO, 337 spines). ns, not significant; Student’s t-test. Scale bar, 500 nm.

Next, we quantitatively analyzed the ultrastructural synaptic organization of the molecular layer by electron microscopy (Fig 3A and 3B). In the middle molecular layer where the dendritic spines of Purkinje cells mainly form synaptic contacts with parallel fiber terminals from granule cells, the density of asymmetric synapses was significantly lower in EFA6C KO mice compared with that in wild-type mice (Fig 3C, WT, 15.8 ± 1.4 / 100 μm^2^; KO, 13.1 ± 0.5 / 100 μm^2^, n = 3 mice of each genotype, *P* = 0.032, Student’s t-test). In contrast, the width of spine heads and the length of the postsynaptic density on asymmetric synapses of Purkinje cells were comparable between the two genotypes (Fig 3D and 3E). To further examine the incidence of free dendritic spines that contain a postsynaptic density but lack synaptic contact with the presynaptic terminal, we performed serial section electron microscopy of the molecular layer from the two genotypes (Fig 4A and 4B). The incidence of free dendritic spines was extremely low in both genotypes and the difference between the groups was not statistically significant (Fig 4C; WT, 0.58 ± 1.02%, n = 330 spines from 3 mice; KO, 0.58 ± 0.52%, n = 337 spines from 3 mice, *P* = 0.989, Student’s t-test).

### Behavioral analyses

**Fig 5:**
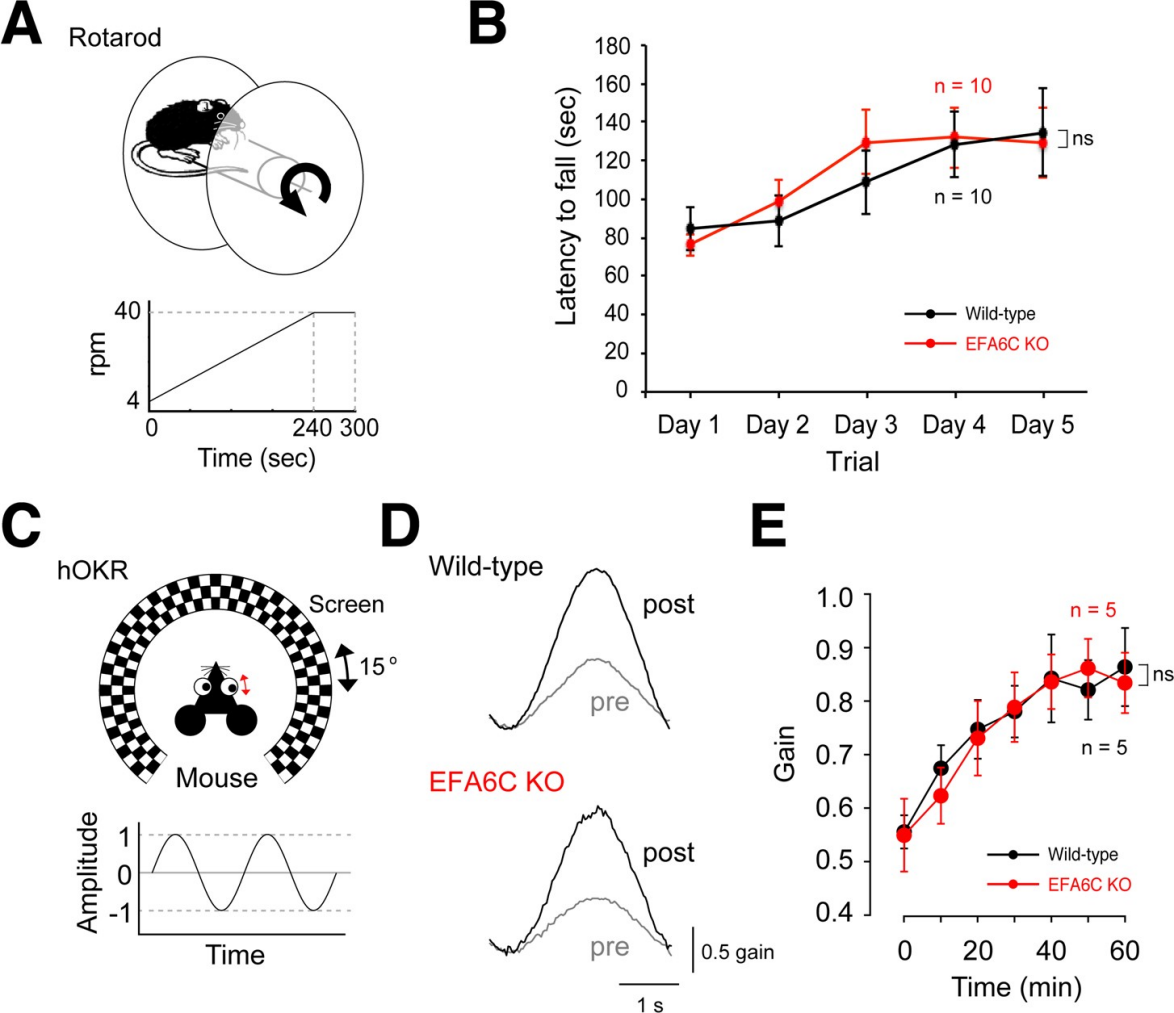
EFA6C KO mice exhibit normal motor coordination and learning. (A) Schematic drawing of the accelerating rotarod test. Ten mice of each genotype were subjected to three daily trials of a rotarod accelerating from 4 to 40 rpm over 4 min for five consecutive days. (B) The rotarod performance showing the lack of significant differences in the latency to falling off the rotarod between the two genotypes. (C) Schematic drawing of the horizontal optokinetic response (hOKR) test. Five mice of each genotype were subjected to a 60-min session of horizontal oscillations of a checked-pattern screen. (D) Representative hOKR waveforms before (pre) and 60 min after (post) training. (E) The OKR performance showing the lack of significant differences in the progression of the adaptation between the two genotypes. The gain was defined as the ratio of the peak-to-peak amplitude of eye movement to that of the screen oscillation. Data are shown as mean ± SEM. ns, no significance using two-way repeated measures ANOVA.

Finally, to examine whether motor coordination and learning were impaired in EFA6C KO mice, mice were subjected to two types of cerebellum-related behavioral paradigms, the accelerating rotarod test (Fig 5A) and the hOKR test (Fig 5C). In the accelerating rotarod test, the latency to falling off the rod consistently increased over 5 consecutive days of three daily trials, without any significant difference between the two genotypes (Fig 4B, *P* = 0.8173, two-way repeated measures ANOVA). In the hOKR, wild-type and EFA6C KO mice displayed adaptation of eye movements tracking the horizontal oscillations of a checked-pattern screen at a comparable level during the 60-min training (Fig 5D and 5E; *P* = 0.8926, two-way repeated measures ANOVA). These results suggest that EFA6C KO mice exhibit normal cerebellum-related motor coordination and learning in these two tests.

## Discussion

Previous studies using primary cultured neurons implicated the EFA6-Arf6 pathway in neuronal processes related to the formation of neural circuits, such as the formation of dendrites [26, 27] and dendritic spines [9] and axonal transport [28]. We have previously shown that three members of the EFA6 family, EFA6A, EFA6C and EFA6D, are abundantly expressed in the adult mouse brain with distinct spatiotemporal patterns [17, 19, 20, 25, 26, 29]. However, the functional significance of individual EFA6 members is unknown at the whole animal level. In this study, we focused on EFA6C, an Arf6 GEF that is enriched in the cerebellum [19], and reported the cerebellar phenotypes of EFA6C KO mice for the first time.

The only significant phenotype found in the cerebellum of EFA6C KO mice was the reduced density of asymmetric synapses on dendritic spines of Purkinje cells, which could be caused by several structural abnormalities in the molecular layer. The present serial section electron microscopic analysis revealed that the incidence of free dendritic spines lacking synaptic contacts was comparably low in both genotypes, excluding the possibility that an increase in free spines lead to the reduced synaptic density as previously reported in mice lacking GluD2 [39] and Cbln1 [50]. Therefore, it is likely that the reduced synaptic density in EFA6C KO mice may be attributable to other deficits in cerebellar structures such as the arborization of dendrites, and the formation and maintenance of dendritic spines of Purkinje cells. Because the EFA6A-Arf6 pathway is implicated in the formation of dendrites and dendritic spine of cultured hippocampal neurons [9, 26, 27], these possibilities need to be tested in future studies.

Contrary to our hypothesis about the importance of the EFA6C-Arf6 pathway in cerebellar development and functions, the cerebellar phenotype in EFA6C KO mice was mild. The lack of significant phenotypes in these mice suggests that EFA6C plays ancillary roles in the cerebellum. Alternatively, it may reflect the existence of various cellular mechanisms to compensate for the ablation of the EFA6C-Arf6 pathway. First, Arf6 can be activated by several Sec7-containing Arf GEFs: EFA6A–D in the EFA6 family [16–18, 20], BRAG1–3 in the BRAG family [34, 36, 44] and cytohesin 1–3 in the cytohesin family [45, 51]. Indeed, Purkinje cells abundantly express EFA6D [29], BRAG2 (our unpublished data), BRAG3 [36] and cytohesin 1–3 [51, 52] as well as EFA6C. Although the present immunoblot analyses failed to detect any compensatory changes in the protein expression of these Arf6 GEFs, it is still possible that their subcellular localization or GEF activities may be changed in Purkinje cells lacking EFA6C. Second, considering the essential nature of actin cytoskeleton remodeling and endosomal trafficking in neurons, there are likely to be crosstalk mechanisms between Arf6 and other small GTPase pathways in neurons. For example, the EFA6A-Arf6 pathway was shown to partly converge on Rac1 to regulate the formation of dendritic spines in cultured hippocampal neurons [9, 11]. In addition, Rab35 was shown to promote nerve growth factor (NGF)-induced neurite outgrowth of PC12 neuroendocrine cells by regulating the activity of Arf6 through the interaction with centaurin-β2/ACAP2, an Arf6 GAP [53]. These findings suggest that Arf6 coordinates with other small GTPases such as Rac1 and Rab35 to regulate neurite formation. Therefore, it is conceivable that such crosstalk mechanisms between Arf6 and other small GTPases may compensate for the lack of EFA6C to maintain the morphology and function of Purkinje cells. Third, there are short alternative isoforms of EFA6A and EFA6D, termed EFA6As and EFA6D1/2s, respectively, which share the C-terminal region containing a PH domain and coiled coil motifs but lack a catalytic Sec7 domain [27, 29]. EFA6As reportedly regulates the dendritic formation of cultured cortical neurons probably through Arf6-independent actin cytoskeleton remodeling [27]. In the present immunoblotting, the anti-EFA6C antibody raised against the C-terminal region of human EFA6C detected an immunoreactive band of 50 kDa in addition to a band for authentic EFA6C. Although the nature of this band is currently unknown, it is possible that a short isoform of EFA6C may exist and function to compensate the lack of EFA6C. Finally, we cannot exclude the possibility that the cerebellar phenotypes may become obvious with aging. For example, mice lacking the prion protein gene showed cerebellar Purkinje cell degeneration, progressive ataxia and impaired motor coordination only after they aged 70–90 weeks [54]. Therefore, follow-up studies are necessary to examine whether EFA6C KO mice develop age-dependent cerebellar phenotypes.

Despite ~17% reduction in the spine density of Purkinje cells, EFA6C KO mice did not exhibit behavioral abnormalities in the accelerating rotarod and hOKR tests. Since ~60% reduction of Purkinje cell synapses is associated with impaired motor functions in mice lacking GluD2 [39], it is possible that the spine reduction in EFA6C KO mice is below the threshold for these motor phenotypes. While ~18% reduction of Purkinje cell spines was reportedly associated with essential tremor in humans [55], neuronal circuits other than parallel fiber-Purkinje cell synapse could be also affected in these patients. Recent functional imaging studies with human subjects and behavioral analyses with genetically modified animals revealed that the cerebellum is engaged not only in motor coordination and learning, but also in higher brain functions such as cognition, perception and emotion [56]. Furthermore, increasing evidence indicates that disturbance in cerebellar development and functions contributes to the pathogenesis of neuropsychiatric disorders such as autism spectrum disorder [57, 58]. Therefore, further comprehensive behavioral analyses are necessary to examine whether EFA6C KO mice exhibit other cerebellum-related behavioral abnormalities such as autistic-like behaviors.

## Supporting information

S1 Fig Sequences analysis of the EFA6C mutant founder mice. The PCR amplicons from genomic DNAs purified from founder mice were subjected to sequencing. The PAM sequence and insertions are shown in red and blue, respectively. Note that two mice (founder #1 and #2) carried a mono-allelic mutation, six mice (founder #3–#8) carried bi-allelic mutations, and two mice (founder #9 and #10) were mosaic with three or four mutant alleles.
(TIF)
