# Supplementary figures and images for: Mice lacking EFA6C/Psd2, a guanine nucleotide exchange factor for Arf6, exhibit lower Purkinje cell synaptic density but normal cerebellar motor functions
Source: PLoS One. 2019 May 16;14(5):e0216960. doi: 10.1371/journal.pone.0216960 (PMC6522047; doi:10.1371/journal.pone.0216960)

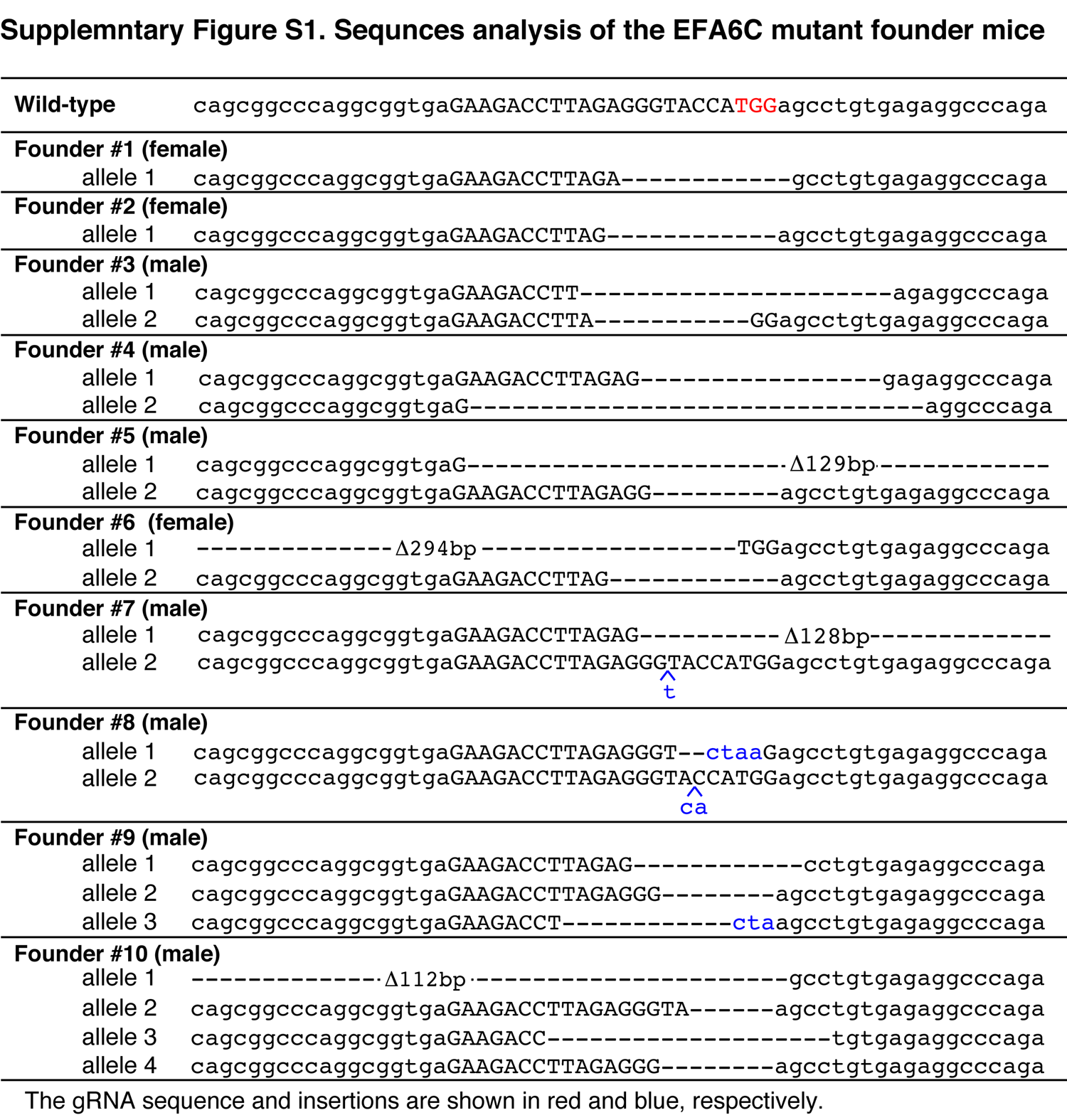

Supplement: S1 Fig — The PCR amplicons from genomic DNAs purified from founder mice were subjected to sequencing. The PAM sequence and insertions are shown in red and blue, respectively. Note that two mice (founder #1 and #2) carried a mono-allelic mutation, six mice (founder #3–#8) carried bi-allelic mutations, and two mice (founder #9 and #10) were mosaic with three or four mutant alleles. (TIF) [file pone.0216960.s001.tif]
